# The land-use land-cover change–emerging infectious disease nexus reconsidered

**DOI:** 10.1093/biosci/biaf045

**Published:** 2025-04-16

**Authors:** Ore Koren, Luis Fernando Chaves

**Affiliations:** Department of Political Science and Ostrom Workshop for Political Analysis and the Tobias Center for Innovation, International Development Indiana University Bloomington, Bloomington, Indiana, United States; Department of Environmental and Occupational Health, School of Public Health-Bloomington, Tobias Center for Innovation, International Development and Department of Geography, Indiana University, Bloomington, Indiana, United States

**Keywords:** One Health, infectious disease, deforestation, economic development, armed conflict

## Abstract

Deforestation due to land-use and land-cover (LULC) change has been linked to increased emerging zoonotic disease risk despite limited local level data on such outbreaks. This Forum reevaluates this risk inference using newly released data on zoonotic disease outbreaks, accounting for Structural One Health features, including socioeconomic development and armed conflict covariates. Event and time series data on disease and forest coverage anomalies at the 0.5-degree level for every month between January 2003 and December 2018 are used to estimate the relationship between LULC and zoonosis using Poisson generalized additive and generalized linear models. Once adjusted for Structural One Health features, outbreak risk is 7%–200% higher in areas that experienced forest cover reversion. These results highlight the importance of accounting for Structural One Health factors when analyzing complex socioecological phenomena such as the LULC–infectious disease nexus.

Anthropogenic behaviors are recognized as key drivers of emerging infectious diseases (Celli 1933, Levins et al. 1994, Brooks et al. 2023). Although the general mechanisms underlying infectious disease emergence are not fully understood, researchers linked deforestation, as a means of shifting land use and land cover (LULC) to increase crop and livestock production, to greater risk of emerging disease (Gottdenker et al. 2014). To test LULC impacts, past studies employed proxies such as species richness or ecological assemblages (Gibb et al. 2020) or used Jones and colleagues’ (2008) emerging infectious disease events database (Murray and Daszak 2013, Loh et al. 2015), which records *n* = 335 emerging infectious disease outbreaks between 1940 and 2004, *n* = 220 of which have geolocated information.

The view arising from this research, however, has tended to narrow the perspective on factors causing the emergence of infectious diseases to merely the changing ecological relations across species, for example, in response to LULC change as if such change was an autonomous force (Plowright et al. 2021). This view ignores the possibility that ecological relations, when they affect human health and well-being, are also social (Levins and Lopez 1999) and political (Bagozzi and Koren 2023, Koren and Bukari 2024). We consider an approach that integrates the study of human, animal (livestock and wildlife), and crop health within an ecosystemic view as One Health (Zinsstag et al. 2012). In practice, One Health is frequently decontextualized by assuming that changes in the ecological relations across species that interact with humans in disease emergence. This despite a large body of evidence showing the impacts of social relations on human biology and its expression in health and well-being indicators (Himmelstein et al. 1990, Rosero-Bixby and Dow 2016, Dickman et al. 2017, Bor et al. 2023, Himmelstein and Woolhandler 2023).

This lack of context has often led to the uncritical acceptance of deforestation as a one-way cause of infectious disease emergence. For example, narratives from decontextualized One Health studies partially drive the Half of the Earth paradigm, which seeks to protect natural lands by restricting human activity over vast areas (Wilson 2016). The Half of the Earth paradigm has been criticized by ecologists, considering biodiversity is clustered in specific areas (Pimm et al. 2018), the wider negative implications for populations engaged in sustainable modes of production that get disrupted by land sparing (Perfecto et al. 2019, Perfecto et al. 2023) and, more generally, by ignoring how uneven economic forces that generate social inequality are deep drivers of biodiversity destruction (Büscher et al. 2017).

A One Health approach provides an advantage over Koch's paradigm that infectious disease is the straightforward expression of an infection by a pathogenic microbe (Kaufmann and Schaible 2005). As we now know, Koch's paradigm is also limited in its ability to even define disease within individual organisms, given the rich diversity of interactions behind microbial pathogenesis and immunity (Tosta 2001). Similarly, at the ecosystem level, a narrowly defined One Health approach to study emerging infectious diseases “has also omitted addressing fundamental structural causes underlying collapsing health ecologies” (Wallace et al. 2015, p. 68). Such structural features, that—when considered within the One Health approach—define Structural One Health, are indicators of how social, political, and economic factors that influence each other and in turn change ecological and evolutionary interactions across pathogens and their hosts. This often produces feedback on the social, economic, and political factors that promote change in the ecologies of pathogens that become recognized as emerging infectious diseases (Levins et al. 1994, Wallace 2009, Wallace 2016).

The Structural One Health approach to understanding disease emergence can be traced back to classical naturalists who studied disease. For example, in the first century CE, Pliny the Elder recognized the relationship between inequities in land tenure and malaria in ancient Rome, a pattern common through most of the documented history of the Agro-Pontino Romano (Celli 1933). This engendered land tenure inequity and endemic malaria transmission that can be reproduced by relatively simple mathematical models (Chaves 2013). Similarly, malaria transmission has been associated with conflict through different historical periods (De Zulueta 1973, 1987, 1994) and currently still affects international relations between states (Bagozzi and Koren 2023). However, current research linking LULC and disease emergence has often failed to acknowledge how LULC change itself is driven by networks of trade, agribusiness development (Bergmann and Holmberg 2016, Ceddia 2020), and other inequality generating features such as armed conflict (Hillesund et al. 2018).

## Evidence from new data

Three empirical limitations of previous research may mean the link between deforestation and emerging diseases is weaker than suggested. First, existing studies that use local outbreak data are limited to specific onsets and are aggregated over years or decades, as in the sparse emerging infectious disease events data set (Allen et al. 2017), or use remote proxies, such as species richness, with the assumption that increased richness implies reduced disease risk (Randolph and Dobson 2012). Second, many LULC studies rely on annual forest estimates and rarely account for the role of seasonality and seasonal changes in vegetation growth, deforestation (i.e., the process by which forest cover is lost), and forest reversion (i.e., the process by which forest cover increases) dynamics. Third, from a Structural One Health perspective, the extant research rarely incorporates wider social, geographic, and economic determinants, including development indicators (Najera 1994, Chaves et al. 2008), modes of agricultural production (Scorza and Rojas 1988, Bergmann et al. 2023), economic inequality (Yamada et al. 2016), relational geographies of uneven ecological exchange in trade and capital circulation (Jorgenson 2006, Chaves et al. 2022), and armed conflict (Iqbal 2006) in their disease risk evaluations. This despite the fact that many regions that are susceptible to emerging diseases are also characterized by low development, inequalities in terms of access to resources, natural resource extractivism, capital dispossession, and protracted conflict (Davis 2001, Harvey 2017, Koehnlein and Koren 2022, Yu et al. 2024, Koren and Bukari 2024).

These issues can hinder outbreak monitoring and response and may also shape LULC policies for reasons that have more to do with sociopolitical dynamics than epidemiological concerns. Therefore, the inclusion of social, geographic, economic, and political conflict indicators in Structural One Health LULC and infectious disease models is necessary for understanding how to alter the underlying conditions that promote transmission. Such analyses can produce insights that will assist reducing both transmission and the risk of new diseases from affecting humans.

In the present forum, we reexamine the LULC and emerging infectious disease relationship with more appropriate estimates of emerging disease outbreaks and disaggregated proxies of seasonal forest growth anomalies, adjusting the results for the role of local-level Structural One Health (socioeconomic, climate, conflict, and seasonal) features. For measuring zoonotic disease outbreaks, we used a new data set of confirmed outbreak events covering 22 emerging zoonotic pathogens from the Geolocated Zoonotic Outbreak Event Dataset (G-ZOD; Koren and Bukari 2024; see the discussion on collection procedures in the supplemental material). G-ZOD includes geolocated information on *n* = 1284 confirmed monthly outbreak events (new and ongoing) in 286 locations in Africa between January 2003 and December 2019 (in contrast to the emerging infectious disease events data set’s *n* = 220 global geolocated outbreaks over six decades) and is measured at the monthly level, as opposed to emerging infectious disease events data set’s annual and multiyear windows (a map of outbreak locations by pathogen type is shown in supplemental figure S1).

These outbreak event data were regressed on a monthly indicator of vegetation growth anomalies (standardized using *Z* scores) within forest areas, which we created using high-quality radar data about forest cover (Shimada et al. 2014, Gao et al. 2020). For analysis, we use AfroGrid, a 0.5-degree grid-month data framework (the recommended spatial and temporal resolution for event data sets such as G-ZOD) that also includes numerous socioeconomic, climatic, and environmental features across the entire continent (Schon and Koren 2022; see supplemental material for a full discussion of our data and methods).

In figure 1, we first plot the bivariate relationship between vegetation anomalies in forest areas and zoonotic disease outbreaks, smoothed using additive modeling, to identify locations and times of high risk. In the next step, we adjusted the bivariate relationship to account for some of the key Structural One Health factors highlighted in extant research (e.g., Levins et al. 1994, Chaves et al. 2008, Wallace 2009)—namely, socioeconomic features (population and economic development), climate conditions (rainfall, temperature, drought), and seasonality, then in addition, for features less mentioned in such research, mainly conflict rates, using the count (Poisson) generalized additive model (GAM) in our full Structural One Health model. Finally, we use a stepwise backward elimination procedure, based on the minimization of the Akaike information criterion (AIC), of the Structural One Health Poisson generalized linear model (GLM) to objectively select covariates and ensure our results are not driven by subjective covariate choices (Faraway 2004).

**Figure 1.**
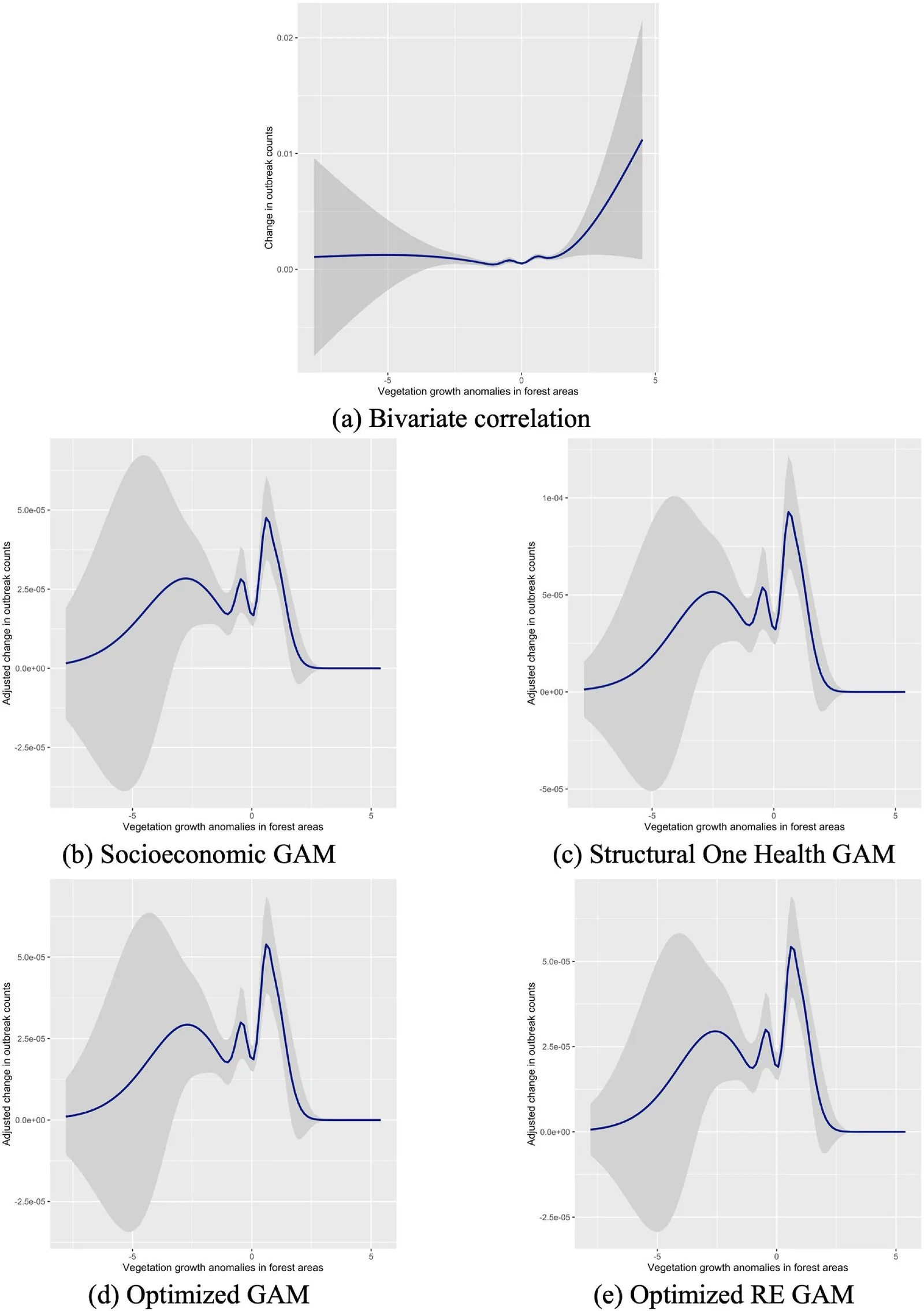
Vegetation growth anomalies in forest areas and zoonotic disease outbreaks in Africa, January 2003–December 2019. (Top) bivariate, (center) Poisson socioeconomic and Structural OneHealth generalized additive model fits, (bottom) backwards elimination optimized Poisson generalized additive model fits. N = 2,171,031, 2,042,991, 1,498,640, and 2,031,029 for the bivariate, socioeconomic, One Health, and optimized models, respectively; the vegetation growth anomalies in forest areas terms in all models are statistically significant (p < .01, two-tailed tests).

Through this process we find that—in line with our claims above—the optimized model include our monthly vegetation in forest area anomalies indicator, in addition to state capacity (population, development, and nonstate conflict) and temperature anomalies, as well as dummies for the spring (May, June, July) and fall (September) months. The resulting best model is also supported by Bayesian model averaging (reported in the supplemental material). We then estimated Poisson GAMs on this optimized specification without and then with random effects by grid cell (our geospatial unit of analysis) and month, in order to account for potential lack of independence, or *pseudoreplication*, in the data (Chaves 2010), given the constrained spatial and temporal nature of the outbreaks we study. Overdispersion tests suggested the data are not overdispersed (*p* = .771, *p* = .750, and *p* = .794 for the socioeconomic, Structural One Health, and optimized models, respectively), thereby confirming the suitability of the Poisson model. The statistical estimates of the four GAMs are reported in supplemental table S2.

Both our parametric GLM and our nonparametric GAM models suggest a complex nonlinear relationship between vegetation growth anomalies in forest areas and disease risk. Starting from the simple bivariate relationship that suggests a monotonic increase in zoonotic disease risk with positive vegetation growth anomalies in forested areas (figure 1a), a result supported by our parametric models (supplemental tables S3–S7). However, the use of nonparametric models suggests that such relationship is nonmonotonic (figure 1b–1e). In general, across all models, extreme negative anomalies for vegetation growth (less than –5 anomalies) are not significantly associated with the emergence of infectious disease (*p* > .05). Unlike the bivariate and parametric models, the nonparametric models suggest that negative mild anomalies, (between –5 and 0 anomalies) are associated with a slight increase in infectious disease emergence risk. However, *positive* anomalies in vegetation growth, conditions that might be associated with forest restoration, increase disease risk when vegetation growth anomalies are between 0 and 1. When vegetation growth anomalies become increasingly positive (more than 1) the risk of infectious disease emergence decreases until becoming not significant for even larger values of vegetation growth anomalies (more than 2.5).

As we adjust for socioeconomics and then for the rest of our Structural One Health features, we find that the association between the positive changes in forest growth anomalies and disease outbreak risk remain, whereas the association between negative forest growth anomalies and infectious disease outbreaks are more uncertain, as illustrated by the greater 95% confidence intervals around the smoothed GAM estimates in figure 1. Most importantly, the shape of the association between positive changes in forest growth anomalies with disease remained unchanged in the optimized models, suggesting that key socioeconomic and security features are crucial in explaining how changes in forest cover affect disease emergence. AIC scores suggest the One Health model (adjusted for all confounders) provides a better fit for the data than the socioeconomic-only adjusted model (AIC_OneHealth _= 12,441.2 and AIC_socioeconomic _= 12,473.4, adjusted for the number of observations), whereas the optimized models provide an even better fit (AIC_optimized _= 12,431.8 and AIC_optimized RE _= 12,422.3, adjusted for the number of observations). Because AIC values are only interpreted comparatively, this suggests that the optimized model with key socioeconomic, conflict, temperature, and seasonal indicators provide the most effective explanation to infectious disease outbreaks in our data set.

We believe that using additional modeling tools to explore our data set could lead to other insights that would be especially valuable if they come from the nuanced and contextualized use of this and other large data sets (Chaves et al. 2023a). Finally, in the supplemental material (section D), we present detailed results where we explore and discuss some of the assumptions for linear models. We found no evidence for a spatial correlation on the basis of the inspection of the Moran's *I* index (Moran 1948) for the residuals (supplemental figure S3) and no evidence for endogeneity and serial autocorrelation using the generalized method of moments models (supplemental table S8). We also found, using Bayesian model averaging, that vegetation growth anomalies in forest areas is as important as key socioeconomic features (population and development), whereas other environmental, climatic, and seasonal features were also important to explain the outbreaks (supplemental table S9).

## Policy discussion

Our results suggest that LULC–infectious disease nexus inferences and the resulting narratives, especially as they inform policy making, need adjustment, with greater attention for Structural One Health features (Wallace et al. 2015). More specifically, several mechanisms may explain the patterns we identify.

One mechanism where positive vegetation growth anomalies increase the risk of infectious disease emergence relates to the return and recovery of populations of potential pathogen reservoir species, such as birds (Matlock et al. 2002) and rodents (Larsson 1977), into areas where forest cover has been reversed. Deforestation reduces these reservoirs’ population size, reducing transmission risk (Dobson 2004, Lloyd-Smith et al. 2012, Faust et al. 2018, Vinson et al. 2022). As the forest cover recovers, however, reservoir populations may also recover and, correspondingly, the number of interactions between humans and reservoir species, which can raise the risk of transmission to humans will increase. Moreover, key species for the regulation of reservoir species might be absent during forest cover recovery (Terborgh et al. 2001), further enhancing the ecological conditions under which populations of reservoir species thrive, and where transmission to humans is exacerbated (Johnson 1965, Tesh et al. 1993). This explanation is ecologically sound, but we also believe that looking at wider Structural One Health contexts can produce more nuanced understandings of why forest reversion can lead to the emergence of zoonotic diseases.

In this case, a second, more intricate mechanism encompasses both reservoir population recovery and the contextual space where pathogen transmission occurs, because several factors interact within the broader biological and social disease emergence environment (Levins 1995). Boundaries and transitional land uses can shape both vector and host distributions and have been historically shown to be critical for the transmission of several zoonotic and vector-borne diseases, for instance via shifting boundaries between agricultural and nonagricultural areas, a transition that often is shaped by economic decisions that externalize potential health costs (Audy 1958, Pavlovsky 1966, Kitron 1998, Lindsay and Birley 2004, Reisen 2010). The influence of factors already studied within disease ecology, such as seasonal changes, can lead to zoonotic disease emergence, both as an expression of biological and climatic patterns that affects forest growth. For example, mathematical modeling shows that seasonality—often modeled using cyclical functions—induces changes in ecosystems, including variations in vegetation growth, floods, and droughts, alongside other ecosystem-wide shifts that can induce changes in vector (e.g., mosquitoes, sand flies) and reservoir hosts (e.g., rodents) behavior, thereby increasing the risk of transmission (Altizer et al. 2006, Salkeld et al. 2023).

However, seasonality—and, more generally, the “natural” environment—can also be shaped by human behaviors. For example, childhood diseases are more likely to be transmitted during the school term (Earn et al. 2000). Similarly, more than any landscape features, modes of agricultural production can predict the burden of disease, as is illustrated by the fact that modes of production were better predictors of COVID-19 burden in the US Midwest prior to the advent of vaccines. Counties engaged in regenerative agricultural practices, geared toward local rather than global consumption and rely on more ecological sound practices for soil and biodiversity conservation, had a reduced number of cases compared with conventional agriculture counties, where food is produced for global markets, and there is less concern about long-term environmental degradation (Bergmann et al. 2023).

Our analysis suggests that pathogen exposure risk may rise during peak harvest seasons, which increases the probability that humans will intersect with wild animal hosts such as mice or insect vectors while working the land, especially in areas close to forest edges (Lemessa et al. 2013). However, the risk of interaction can be modulated by the well-being of the humans engaging in these activities, with poorer populations being generally more likely to be infected or exposed to pathogens (Chaves et al. 2008). Similarly, conflicts in rural developing areas can also intensify during the harvest season, because armed groups attack civilians to appropriate crops, cattle, and cash (Ubilava et al. 2023) and may push civilians to seek refuge into the forest, where they are exposed to greater risk of catching zoonotic pathogens from wildlife reservoirs or through the bite of infected vectors. This situation is exacerbated by long conflicts, which induce displacement, with resettlement often occurring at the edges of forest areas (Salemi 2021), where forest recovery can lead to increased transmission rates. Beyond seasonality, zoonotic disease emergence is also driven by broader socioeconomic determinants.

For example, past work has shown how *cutaneous leishmaniasis*—a disease whose emergence was paradigmatically assumed to be driven by deforestation (Herrer and Christensen 1976)—is clustered in areas with high levels of social exclusion, with forest cover modulating the impacts of climatic anomalies, but not being—in and of itself—a major determinant of the disease in tropical settings (Chaves et al. 2008). Other studies show that, by inducing population movement and increasing socioeconomic disparities, trade flows may have affected the spread of diseases such as COVID-19 in Mesoamerica and the Caribbean (Chaves et al. 2022) and Ebola in West Africa (Wallace et al. 2016, Perfecto et al. 2023).

In line with the view of zoonotic disease emergence as a biological and social phenomenon, our local monthly estimation approach illustrates the weakness of proposing a one-directional link between LULC and emerging infectious disease risk, a conclusion echoed by mathematical models linking land-use dynamics and transmission (Wells et al. 2019, Vinson et al. 2022) and studies of specific diseases within nuanced contexts (Yamada et al. 2016). Granted, we also find deforestation is associated with disease emergence, underscoring the need to also address connections between biodiversity conservation and disease emergence, all while seeking to better understand how land sparing and land dispossession affect human well-being, leading to population displacement, conflict, and disease emergence (Davis 2001, Kremen 2015, Gottdenker and Chaves 2024). However, the claim that biodiversity conservation can only be achieved through land sparing is unsupported by our findings. Land dispossession can actually harm socioeconomic development and intensify conflict, thereby leading—per our findings—to an even higher risk of emerging infectious disease outbreaks (Froese and Schilling 2019). Both conflict and disease protract in underdeveloped regions, where the state is weak, development levels are low, and—often—warfare has been ongoing for years (Davis 2001, Koehnlein and Koren 2022, Bagozzi and Koren 2023).

Accordingly, we believe both researchers and policymakers should reconsider their views and incorporate Structural One Health features that account for the heterogeneity of landscapes and social formations where diseases emerge into assessments of the LULC–infectious disease nexus. Paradigms that view agrarian–ecological matrixes as critical for biodiversity conservation while incorporating the role of conflict and socioeconomic factors will be helpful in preventing disease emergence, ensuring policy burden does not disproportionally fall on Indigenous and other local vulnerable populations, currently affected by land dispossession in the name of biodiversity conservation (Büscher et al. 2017, Chaves et al. 2023b).

Finally, this study is a step in improving our understanding of zoonotic disease emergence as a complex socioecological phenomenon. Our results are—in the current state—open to future extensions that further test our hypothesis and validate our results, mainly the need to pursue a Structural One Health that more closely looks at the social, geographic, economic, and political dynamics that interact with the biological agents that create disease. As Levins (2010, p. 48) warned, “a problem has to be posed large enough to fit a meaningful solution”; moving to a Structural One Health approach can only help us to better understand the widely defined changing ecological relations driving infectious disease emergence. The present study launches a new research trajectory to explore more basic ideas surrounding the important nexus between LULC and emerging infectious diseases and can help, in line with the recommendations of past research (Celli 1900, Levins et al. 1994, Brooks et al. 2023), more effectively shift interventions in ways that can facilitate disease prevention.

## Supplementary Material

biaf045_SIFileBioS_11_24_24F

## Author Biography

Ore Koren (okoren@iu.edu) is an associate professor in the Department of Political Science and affiliated faculty at the Ostrom Workshop for Political Analysis and the Tobias Center for Innovation in International Development Indiana University Bloomington, in Bloomington, Indiana, in the United States. Luis Fernando Chaves (lfchavs@gmail.com) is an associate professor in the Department of Environmental and Occupational Health, in the School of Public Health-Bloomington, in the Tobias Center for Innovation in International Development and in the Department of Geography at Indiana University, in Bloomington, Indiana, in the United States.
