# Supplementary material for: The land-use land-cover change–emerging infectious disease nexus reconsidered
Source: Bioscience. 2025 Apr 16;76(10):1171–7. doi: 10.1093/biosci/biaf045 (PMC13702796; doi:10.1093/biosci/biaf045)
Supplement: Supplementary file 1 [file biaf045_sifilebios_11_24_24f.docx]

Supporting Information (SI) for

**The Land Use Change-Emerging Infectious Disease Nexus Reconsidered**

**Table of Contents**

| **A. Geolocated Disease Outbreak Event Data** | 2 |
| --- | --- |
| **B. Data and Methods** | 6 |
| **C. Main Model Estimates and Robustness Models** | 10 |
| **D. Empirical Limitations and Sensitivity Analyses** | 16 |
| **E. References** | 20 |

**A.	Geolocated Disease Outbreak Event Data**

Data on infectious disease outbreaks in Africa between January 1996 and December 2019 was taken from the Geolocated Zoonotic Disease Outbreak Event Dataset (G-ZOD; Koren and Bukari 2024), although due to limited availability on NDVI measures used to calculate vegetation anomalies in forest areas, the analysis is constrained to the Jan. 2003 – Dec. 2019 period. G-ZOD records individual outbreaks under a location-date “where-and-when” framework, coding, first and foremost, information on (i) pathogen, (ii) province/state, and (iii) date/month and year, and (iv) credible source, as well as (based on data availability) information on: (iv) the number of people affected by and (v) the number of people who died from the pathogen; (vi) the exact date and (vii) exact location of the outbreak; (viii) the cause of infection (farm animals, wildlife, person-to-person, or other); and (ix) additional comments. The 22 pathogens included in G-ZOD were all reported by the WHO as pathogens of concern for African states (WHO 2023), and are:

| 1. Ebola-Zaire 2. Ebola-Sudan 3. Ebola-Täi forest 4. Ebola-Bundibugyo 5. Ebola-Reston 6. Marburg 7. Yellow fever 8. Rift Valley fever | 9. H1N1 10. H5N1 11. SARS 12. MERS 13. Chikungunya 14. Lassa 15. Dengue 16. Monkeypox | 17. Septicaemic plague 18. Bubonic plague 19. Crimean-Congo hem. fever 20. Zika/Zica 21. Anthrax 22. Rabies |
| --- | --- | --- |

Search for additional pathogens – including H9N1, H7N9, H5N6, SARS-COV-19, and shigellosis – was also conducted, but no outbreaks involving these pathogens were identified over the period in any African state. For illustration, a map summing confirmed outbreaks by location is reported in Figure S1. Note that in this map we collapsed some categories – e.g., Ebola and swine flu – other, to save space. Additionally, some of these pathogens did not experience an outbreak during this curtailed period.

**Fig. S1.** Zoonotic disease outbreaks in Africa by type and exact location, Jan. 2003 – Dec. 2019, with JAXA % forest coverage (by 0.5-degree grids) estimates.

G-ZOD defines an outbreak as a local incident involving one of the pathogens mentioned above, where at least one individual was affected (Koren and Bukari 2024). Each of 1,362 confirmed outbreak events between Jan. 1996 and Dec. 2019 (or *n*=1,284 events between Jan. 2003 and Dec. 2019) in G-ZOD (note: G-ZOD record several dozens of additional incidents that were unconfirmed, which are *not* part of the *n*=1,362, as well as country level incidents that were also not included in these specific data) was recorded in four stages:

1. Identifying an outbreak event for a pathogen of interest: using the LexisNexis Uni database, all outbreak events in Africa involving the 22 pathogens of interest were identified (as mentioned above, searches for additional pathogens was also conducted). As mentioned above, an outbreak event was coded if it affected at least one person. For the geospatially coded data, events that could be classified to the village, or at the very least to the province (1^st^ admin), month and year level was retained.
2. Coding relevant information on the outbreak: after identifying relevant incidents, information on different aspects of the outbreak event (e.g., number of people affected, dead, etc.) was coded using reports in reputable English language sources (e.g., NYT, Associated Press, BBC, Reuters, CNN, and CBS). For illustration, Fig. S2 (top plot) shows how information is obtained from a news story. All news stories were downloaded, given the incident’s unique ID, and stored for future replication.
3. Confirmation using duplicates: Most outbreaks included more than one media report. For instance, a report might mention outbreak in one village, and a later report will then mention outbreaks in several villages, including the one from the previous report. Our team combined information from different reports to get the best estimates for the information in (2).
4. Triangulating outbreak event data with ISID and WHO reports: after coding information on each outbreak, each incident was triangulated for confirmation twice, once using the ProMed mail database (ISID 2023), which only report laboratory confirmed outbreaks; and separately using WHO disease outbreak news, which report outbreaks confirmed by a government official or agencies like the CDC. Figure S2 (bottom plot) also illustrates how the same incident coded using the story from the upper plot was confirmed. Each of the incidents used in constructing the figure in the main note was confirmed by at least one of the two sources (ProMed/ISID or WHO).

**Fig. S2:** A story of an Ebola Zaire outbreak (top) and WHO confirmation of the story (bottom)

**B.	Data and Methods**

*Data and the unit of analysis*

All data and variables are aggregated into AfroGrid,^15^ where the unit of analysis is the 0.5-decimal degree grid cell (square cells of 55km x 55km at the equator) month for Jan. 2003 – Dec. 2019. A key advantage of the 0.5-degree level for our purposes is that it is the highest resolution geospatial unit recommended when event-based data such as the G-ZOD events and conflict incidence measures used here (Weidmann 2015). Briefly, event-based data collection efforts (as discussed above) rely on media reporting. Especially in remote areas, village names might be switched or mispronounced, or reporters won’t be able to access the exact site, which – in contrast to more precise outbreak investigations – creates some ambiguity as to the exact village or location of the event. Such ambiguities are resolved once the data is aggregated to the 0.5-degree level. The event-data approach provides for much coarser granularity of confirmed outbreaks, although the reliance on event data (as discussed above) means that the recommended resolution for an effective analysis (even though information on exact location is available in the vast majority of cases) is lower. Nevertheless, considering the cross-continental focus, the 0.5-degree resolution is still very high – there are 10,674 African 0.5-degree grid cells observed monthly over the Jan. 2003 – Dec. 2019 period, for a total of 2,177,496 panel observation, although we lose some observations in the socioeconomic and full model plots due to the limited availability of information on some of the controls (e.g., limited population in the Sahara Desert). Summary statistics for all variables are in Table S1.

**Forest growth anomalies:** This grid-month variable was constructed in three steps. First, we identified all 0.5-degree grid cells with at least 10% forest and jungle area coverage according to the Forest/non-forest Map (FNF) land classification based on Phased Array L-band synthetic aperture radar (PALSAR) images by The Japan Aerospace Exploration Agency (JAXA) (20% of all African grid cells) (Shimada et al. 2014), which is considered state-of-the-art in measuring forest areas. The resolution of this product is 25m, but images were reprojected to have a 0.5 resolution using the Google Earth Engine in order to be compatible with other datasets available in AfroGrid. Information on forest/non-forest areas was available for 2008-2010 and 2015-2017 and was extrapolated for each year in the data using the last-value-carried-forward approach, with missing values for 2003-2007 extrapolated from the 2008 dataset. In the second step, we used AfroGrid’s existing monthly Normalized Difference Vegetation Index (NDVI) (Busetto and Ranghetti 2016) indicator to code the vegetation growth (i.e., the extent of the grid cell’s vegetation coverage at any given month between Jan. 2003 and Dec. 2019) within each of these forest-0.5-degree grid cells. To measure the degree of change/variability of vegetation within forest areas more specifically, in the third step, we calculated NDVI anomalies over the entire series as done in past research (Koren and Schon 2023). Here, we first calculated the mean and standard deviation of NDVI for each African grid cell over the entire Jan. 2003 – Dec. 2019 period. We then calculated anomalies in vegetation anomalies in forest areas as standardized *Z* scores measuring the magnitude of the change using the formula: (NDVI_it_ – mean NDVI_i_)/ Standard deviation NDVI_i_ (where the subscripts *i* and *t* correspond to 0.5-degree grid cell and month, respectively).

A key advantage of this indicator is that standard forest/land use measures– which, while appropriate for phenomena at higher spatial resolution, are only estimated annually/multi-annually – it allows us to measure *monthly* variations in vegetation within forest areas. NDVI data are generated at 16-day intervals at 0.05-degree level, and were aggregated by AfroGrid to the 0.5-degree level by summing the total level of vegetation coverage in each grid cell *i* during a given month *t* based on interval-average estimates, to correspond to the event data issues mentioned above. The importance of accounting for seasonality in research on biomass shifts has been highlighted by past studies (Gao et al. 2020). In these regards, our approach of using an NDVI-based measure to approximate seasonality in forest areas follows established practices in the extant research, which similarly used NDVI based measures to approximate seasonal disturbances in forest and forest-boundary areas (Verbesselt et al. 2012, Kuenzer et al. 2015).

**Zoonotic disease outbreak events:** These data are described in detail in Section A.

**Nighttime light:** This indicator of local development and state capacity (Koren and Sarbahi 2018) measures the total number of pixels with illumination within a 0.5-degree cell during a given year based on the DMSP-OLS system, corrected using the VIIRS correction method (Li et al 2020) and included in AfroGrid. Due to the range of this variable, it was log(x+1) transformed. A key advantage of this nighttime light measure is that – due to its reliance on the more sensitive VIIRS data for calibration – it has much higher sensitivity then DMSP-OLS data, which is especially crucial when studying remote developing areas, which are also susceptible to disease outbreaks (and which represent a high share of the 0.5-degree cells in our sample). At the same time, this measure overcomes the limited temporal availability of VIIRS data, providing a longer time series.

**Population densities:** An indicator to approximate annual population densities within each African grid cell based on data from the World Population Counts (WorldPop) dataset, which estimates 0.08 degree (1 km) annual population data between 2000 and 2020 using census data (Tatem 2017, Lloyd et al 2019, Yin et al. 2021). Due to the range of this variable, it was log(x+1) transformed. WorldPop provides a major advantage over other datasets (e.g., GPW V.4) when it comes to analyzing population impacts in remote and rural developing areas (which represent a high share of the 0.5-degree cells in our sample). Specifically, WorldPop is ideal for situations where: “it has either been many years since the last census and simple projections from this baseline may be highly uncertain, or the most recent census did not cover the entire country due to inaccessibility/insecurity,” or in cases where the researcher is specifically “interested in global/continental coverage and/or changing population numbers over time,” as well as in cases where the research requires population totals to match census counts, official projections or UN estimates at national or sub-national scales” (WorldPop 2023). WorldPop solves these issues by combining both “top-down” and “bottom-up” data, which allows for the creation of a comprehensive annual series, and which addresses limited availability in rural, underdeveloped, and conflict afflicted regions.

**State conflicts:** This count indicator measures the total monthly number of conflict events initiated by state actors (e.g., military), including civil war events, in a given 0.5-degree cell from the Geolocated Event Dataset (GED; Sundberg and Melander 2013). Due to the range of this variable, it was log(x+1) transformed. GED is considered the state of the art when it comes to geolocated civil war event data and is hence used here (Eck 2012).

**Nonstate conflicts:** This count indicator measures the total monthly number of conflict events initiated by nonstate actors (e.g., rebels, militias, vigilantes), including civil war events, in a given 0.5-degree cell from the Geolocated Event Dataset (GED). Because nonstate conflicts proliferate where the state has no presence, it is an indicator of state capacity, similarly to nighttime light emissions and population densities. Due to the range of this variable, it was log(x+1) transformed.

**Precipitation anomalies:** This variable, created by AfroGrid, measures normalized (in *Z* scores) monthly anomalies in rainfall within each 0.5-degree cell over 30 years using data from CRU TS monthly high-resolution gridded multivariate climate dataset (Version 4) (Harris et al. 2020).

**SPEI (drought):** This variable, created by AfroGrid using the Standardized Precipitation Evapotranspiration Index (SPEI) (Beguería et al. 2010) for each 0.5-degree grid cell and month in the data. SPEI is a drought indicator of the difference between precipitation and the potential evapotranspiration index for a given time scale, and as a result, is considered more accurate than measures that rely only on water deficits or rainfall to measure drought (Koren and Schon 2023).

**Temperature anomaly:** This variable, created by AfroGrid, measures normalized (in *Z* scores) monthly anomalies in temperature within each 0.5-degree cell over 30 years using data from CRU TS monthly high-resolution gridded multivariate climate dataset (Version 4).

*Methods*

Our modelling strategy relies on combination of (generalized) additive and linear modeling. The reliance on Poisson models with fixed effects is a standard approach in econometric modeling for both parametric non/semi-parametric methods. Our identification relies on the following equations:

$y_{it}=\alpha+f\left( r_{it} \right)+ \epsilon_{it}$	(1)

$y_{it}=\alpha+f\left( r_{it} \right)+\beta_{1}n_{it}+ \beta_{2}p_{it}+ \epsilon_{i}$	(2)

$y_{it}=\alpha+f\left( r_{it} \right)+\beta_{1}n_{it}+ \beta_{2}p_{it}+ \beta_{3}s_{it}+ \beta_{4}ns_{it}+ \beta_{5}r_{it}+ \beta_{6}d_{it}+ \beta_{7}h_{it}+\boldsymbol{m}_{\boldsymbol{t}}+ \epsilon_{i}$	(3)

$y_{it}=\alpha+f\left( r_{it} \right)+\beta_{1}n_{it}+ \beta_{2}p_{it}+ \beta_{3}{ns}_{it}+ \beta_{4}h_{it}+\beta_{5}m5_{t}+ \beta_{6}m6+ \beta_{7}m7_{t}+ \beta_{8}m9_{t}+\epsilon_{it}$	(4)

Eq. 1 is a simple smoothed bivariate correlation. Eq 2. adjusts the estimates for socioeconomic development (nighttime light and population) and 0.5-degree grid cell heterogeneities. Eq. 3 then additionally adjusts the estimates for conflict, climate, and seasonal controls. Eq 4. only keeps the indicators selected by the stepwise backward elimination procedure. y_it_ is a vector measuring the monthly count of confirmed outbreak events; r_it_ is a measure of vegetation anomalies forest areas in a given 0.5-degree cell in a given month; n_it_ is a vector measuring annual nighttime light emissions; p_it_ is a vector of annual population densities; s_it_ is a count vector of monthly state-initiated conflicts; ns_it_ is a count vector of monthly nonstate-actor initiated conflicts; r_it_ are monthly precipitation anomalies; d_it_ is a monthly measure of water deficits (SPEI/drought); h_it_ are monthly temperature anomalies; **m_t_** is a vector of fixed effects for each month accounting for seasonal trends (with January being treated as the reference category); m5_t_, m6_t_, m7_t_ and m9_t_ are dummy variables for May, June, July, and September, respectively; and *ε*_it_ are standard errors and *ε*_i_ are standard errors pooled by 0.5-degree grid cell to account for heterogeneities (in the GLM models *ε*_i_ is standard errors clustered by grid cell). *f*() is a smoothing function that uses best fitting/least penalized cubic regression splines; and β are each independent variable’s respective coefficient for the parametric variables. The Optimized RE model builds on Eq. 4, but adds smooth random effect terms for both grid cell and month. These random effects account for lack of independence in the data (Chaves, 2010) that might arise spatially, for example from closer grids being more similar to each other, or temporally from grids having autocorrelated events.

In the generalized linear model (GLM) versions, we replace the smoothing function with a parametric treatment (β coefficient). We calculate smoothed (GAMs) by holding all control variables at their modes for binary variables, medians for ordinal variables, or means for continuous variables. Model estimates for Fig.1 are in Tab. S2,. As mentioned in the main text, overdispersion tests (*p*= 0.771, *p*= 0.750, and *p*= 0.794 for the socioeconomic, OneHealth, and Optimized models, respectively) confirm the use of the Poisson model. Nevertheless, for robustness, we estimated several sets of linear (ordinary least squares) and zero-inflated models in Tables S3 – S5. All analyses were conducted using the statistical software R. In the generalized methods of moments (GMM), we use shallow lags of the dependent variable (*t*-2 to *t*-8) as recommended by extant research (Roodman 2009) and two-way effects to identify the directional effect flowing from vegetation anomalies in forest areas to zoonotic outbreaks, accounting for serial correlation. With the two-way effects, an equation is estimated for each grid cell over time, the grid cell and month fixed are effectively rendered redundant and flushed out, providing a straightforward estimator.

**Tab. S1:** Summary Statistics

**C.	Main Model Estimates and Robustness Models**

**Tab. S2.** Poisson GAM Estimates of Zoonotic Disease Outbreaks

**Tab. S3.** Poisson GLM Estimates of Zoonotic Disease Outbreaks

**Tab. S4.** Additive Model Estimates of Zoonotic Disease Outbreaks

**Tab. S5.** Linear Model Estimates of Zoonotic Disease Outbreaks

**Tab. S6.** Linear Model Estimates of Zoonotic Disease Outbreaks with Country Fixed Effects

**Tab. S7.** Parametric Zero-Inflated Model Estimates of Zoonotic Disease Outbreaks

**D.	Empirical Limitations and Sensitivity Analyses**

It is important to recognize some potential limitations of this empirical setting. First, while the level of resolution (0.5-degree grids) is arguably sufficiently high to address spatial correlation in outbreak reporting (Weidmann 2015), there is the possibility that spatial correlation is explaining the results, as these issues are not directly accounted for in the models above. We calculated the Moran’s index (I) using residuals from a temporally collapsed model with a balanced dataset (i.e., where cells with missing information were omitted) by summing the total outbreaks for each grid cell over the study period. For calculating the contingency matrix, we relied on the widely used Queen neighbors (Moran 1948; Chen 2021). We find that the Moran I statistic is 0.017, compared with a mean of outbreaks per cell of 0.156 (robust standard errors do not meet the statistical significance threshold of 0.05, two-tailed tests, widely used in the social sciences). The spatial correlation is reported in Figure S3.

A second concern relates to the role of endogeneity (i.e., that disease outbreaks could impact forest reversion, e.g., if people leave villages and forest grow again) and serial correlation or autocorrelation (e.g., outbreaks might be more likely in the same location they happened before). To empirically test if our results are sensitive to this issue, we estimate a set of two-way difference generalized method of moment (GMM) models, which are specifically designed to handle exactly these concerns in panel data (Arellano and Bond 1991). These models leverage past values of the dependent variable to exogenize contemporaneous variations in outbreaks and, due to the use of two-way effects, isolate and account for endogeneity and serial correlation within each specific grid cell for which data is available. Due to the significant computational demands of these models, we had to divide our sample into three portions (2014-2018, 2008-2013; and 2003-2007). As Tab. S8, shows, across all three samples, we find the vegetation growth anomalies in forest areas coefficient maintains its sign and statistical significance, suggesting these issues are unlikely to be driving the results.

Finally, we are aware that, while relying on relevant literature to select our controls, we still made subjective choices pertaining to covariate selection, which could influence the point estimates obtained from our empirical exercise. To this end, note that the optimized models, which rely on stepwise backward eliminations procedure to identify to most important indicators, should address this concern empirically. Nevertheless, to further whether this is an overriding concern, in Table S7, SI File, we report estimates from a Bayesian Model Averaging (BMA) approach applied to our count models (Montgomery and Nyhan 2010). Briefly, BMA evaluates the probability (0 to 1) that a given predictor is included in the “best” models. We find that while measures of weak state capacity indicators (nighttime light, population, nonstate conflict) are the most important (pr=0.98-0.99), vegetation growth anomalies in forest areas is the next most important predictor (pr=0.46), and as such, has a clear substantive impact on increasing the rate of zoonotic outbreaks, greater than that of all environmental and climate variables, for example, which holds regardless of the controls we chose to include.

**Figure S3.** The relationship between outbreaks and their spatially lagged counterparts. The Moran’s I coefficient (captured by the slope of the trendline) is 0.017 (*p*= 0.06, based on robust standard errors and a two-tailed test).

**Tab. S8.** Two-Way Difference GMM Models of Zoonotic Disease Outbreaks

**Tab. S9.** Bayesian Modeling Averaging Probability Estimates

**E.	References**

Beguería, S., Vicente-Serrano, S. M. & Angulo-Martínez, M. A multiscalar global drought dataset: the SPEIbase: a new gridded product for the analysis of drought variability and impacts. *Bulletin Am. Met. Society* 91, 1351–1354 (2010).

Busetto, L. and Ranghetti, L. Modistsp: An R package for automatic preprocessing of MODIS land products time series. *Computers & geosciences* 97, 40–48 (2016).

Chaves, L. F. An Entomologist Guide to Demystify Pseudoreplication: Data Analysis of Field Studies With Design Constraints. *Journal of Medical Entomology* 47, 291-298 (2010)

Eck, K., 2012. In data we trust? A comparison of UCDP GED and ACLED conflict events datasets. *Cooperation and Conflict*, 47(1), pp.124-141.

Gao, Y., Skutsch, M., Paneque-Gálvez, J. and Ghilardi, A., 2020. Remote sensing of forest degradation: a review. *Environmental Research Letters*, *15*(10), p.103001.

Harris, I., Osborn, T. J., Jones, P. & Lister, D. Version 4 of the CRU TS monthly high resolution gridded multivariate climate dataset. *Sci. Data* 7, 1–18 (2020).

International Society of Infectious Disease (ISID). 2023. “ProMED Mail.” Last access, May 26, 2023. https://promedmail.org.

Koren, O., and Bukari, K. 2024. (Re)Emerging Disease and Conflict Risk in Africa, 1997 – 2019. Accepted in Principal at *Nature Human Behaviour*.

Koren, O. and Sarbahi, A.K., 2018. State capacity, insurgency, and civil war: A disaggregated analysis. *International Studies Quarterly*, *62*(2), pp.274-288.

Koren, O. and Schon, J., 2023. Climate change, cash crops, and violence against civilians in the Sahel. *Regional Environmental Change*, *23*(3), p.112.

Kuenzer, C., Dech, S. and Wagner, W., 2015. *Remote sensing time series revealing land surface dynamics: Status quo and the pathway ahead* (pp. 1-24). Springer International Publishing.

Li, X., Zhou, Y., Zhao, M. & Zhao, X. 2020. A harmonized global nighttime light dataset 1992–2018. *Sci. Data* 7, 1–9.

Lloyd, C. T. et al. 2019. Global spatio-temporally harmonised datasets for producing high-resolution gridded population distribution datasets. *Big Earth Data* 3, 108–139.

Roodman, D., 2009. A note on the theme of too many instruments. Oxford Bulletin of Economics and statistics, 71(1), pp.135-158.

Shimada, Masanobu, Takuya Itoh, Takeshi Motooka, Manabu Watanabe, Shiraishi Tomohiro, Rajesh Thapa, and Richard Lucas. 2014. "New Global Forest/Non-forest Maps from ALOS PALSAR Data (2007-2010)", *Remote Sensing of Environment* 155,13-31. DOI=10.1016/j.rse.2014.04.014.

Sundberg, R. and Melander, E. 2013. Introducing the UCDP georeferenced event dataset. *J. Peace Res.* 50, 523–532.

Tatem, A. J. 2017. Worldpop, open data for spatial demography. *Sci. Data* 4, 1–4.

Verbesselt, J., Zeileis, A. and Herold, M., 2012. Near real-time disturbance detection using satellite image time series. *Remote Sensing of Environment*, *123*, pp.98-108.

Weidmann, N.B., 2015. On the accuracy of media-based conflict event data. *Journal of Conflict Resolution*, *59*(6), pp.1129-1149.

World Health Organization (WHO). 2023. “Disease Outbreak News (DONs).” Last accessed, May 30, 2023. <https://www.who.int/emergencies/disease-outbreak-news>.

WorldPop. 2023. WorldPop gridded population estimate datasets and tools. How are they different and which should I use? Accessed Aug. 3, 2023. <https://www.worldpop.org/methods/populations/>.

Yin, X., Li, P., Feng, Z., Yang, Y., You, Z. and Xiao, C., 2021. Which gridded population data product is better? Evidences from mainland southeast Asia (MSEA). *ISPRS International Journal of Geo-Information*, *10*(10), p.681.
